# Evaluation of the effectiveness and tolerance of tetracosactide in the treatment of post-dural puncture headaches (ESYBRECHE): a study protocol for a randomised controlled trial

**DOI:** 10.1186/s13063-019-4015-y

**Published:** 2020-01-08

**Authors:** Célia Depaulis, Nadia Steer, Léa Garessus, Dominique Chassard, Frédéric Aubrun

**Affiliations:** 1 Hospices Civils de Lyon, Hôpital Femme Mère Enfant, Lyon, France; 2 Hospices Civils de Lyon, Hôpital de la Croix Rousse, Lyon, France; 3 Université Claude Bernard Lyon 1, HESPER EA 7425, Lyon, France

**Keywords:** Post-dural puncture headache, Tetracosactide, Cosyntropin, Postpartum, Blood-patch, Epidural

## Abstract

**Background:**

Post-dural puncture headache (PDPH) is one of the most common complications of neuraxial anaesthesia. It limits patients’ general activity and increases the length of hospital stays and the use of care. It is particularly disabling during the postpartum period, when mothers have to take care of their child. Epidural blood patch is the standard treatment for PDPH. However, it is an invasive procedure that may result in rare but serious complications. Recent evidence has suggested that adrenocorticotropic hormone (ACTH) is effective in the management of PDPH. The aim of this study is to assess the efficacy and safety of tetracosactide (Synacthen®), a synthetic analogue of ACTH, for PDPH treatment in patients who receive neuraxial anaesthesia during labour.

**Methods:**

This randomised, double-blind, placebo-controlled, parallel-arm trial, is performed in two French university hospitals. Eligible patients are those suffering from postpartum PDPH, who are randomised to receive either 1 mg of tetracosactide intravenously over 20 min or to 0.9% saline (placebo). The primary endpoint is the rate of epidural blood patch within a 15-day follow-up period. Headache duration, pain intensity, reduction of general activity, increase in length of hospital stay, adverse events, analgesic use (type and duration) and number of blood patches per patient in each group are recorded.

**Discussion:**

We expect a decrease in the use of epidural blood patch in those receiving tetracosactide, thus indicating a decrease in PDPH symptoms in these patients. This will define the therapeutic success of tetracosactide and the possibility to use this treatment as a non-invasive alternative to blood patch for PDPH treatment.

**Trial registration:**

*Primary Registry*

ClinicalTrials.gov Protocol Registration and Results System

Date of Registration 24 June 2016

Unique Protocol ID 69HCL15_0429

*Secondary IDs*

EudraCT Number 2015–003357-17

ClinicalTrials.gov ID NCT02813655

ANSM 160214A-31

**Protocol version**

V4 28/09/2018

## Background

Epidural analgesia is the most effective method to control pain during labour and is frequently used in France (77% of vaginal deliveries in 2010) [1]. For caesarean sections, artificial deliveries, or uterine revisions, spinal anaesthesia is preferred to general anaesthesia due to the risk associated with the latter in pregnant women (such as difficult management of airway) [2]. However, neuraxial anaesthesia can also lead to adverse effects, in particular, post-dural puncture headache (PDPH). The incidence of PDPH following dural puncture mainly depends on the type of needle used (diameter, bevel) [3–10]. For instance, the incidence is reported to be between 0.7% and 4% when 24–27 G conical needles are used for spinal anaesthesia and between 50% and 85% when large Tuohy needles are used for epidural analgesia [3, 10, 11]. Since the incidence of dural puncture during epidural placement is reported to range from 0.04 to 6% [12], the incidence of PDPH among patients receiving these type of anaesthesia (spinal and epidural) is actually similar. In addition, postpartum women are more prone to PDPH than the general population [13–15].

PDPH is a clinical diagnosis. According to the International Headache Society, it is a severe postural, bilateral, disabling, constrictive, occipital or diffuse headache that radiates to the neck and develops within 7 days after the dural puncture. In 66% of cases, it occurs during the first 48 h, and in 90% of cases, during the first 3 days [3]. PDPH is aggravated by a sitting or standing position and is relieved by lying down. Other symptoms such as neck stiffness, hearing disorders (tinnitus, hypoacusia) [16], dizziness, photophobia, nausea or vomiting, and diplopia by injury of the cranial nerve IV [17] may be associated with the headache, and patients remain afebrile. Differential diagnoses are migraine, pre-eclampsia, meningitis, intracranial haemorrhage, cerebral thrombosis, pneumocephalus and tension headaches [18, 19].

As described by Vandam and Dripps, as early as 1956 [20], the main cause of PDPH appeared to be intracranial hypotension by leakage of cerebrospinal fluid (CSF). CSF is critical in reducing impacts between the brain and the cranium during orthostatism, and the loss of this cushioning leads to headaches. Furthermore, the cerebral and meningeal vessels are stressed by the downward displacement of the brain during orthostatism. This caudal traction activates the stretch-sensitive receptors of the trigeminal nerves (frontal headache), glosso-pharyngeal and vagus nerves (occipital headache) [11, 21, 22], and the first three cervical nerves (arm pain or neck pain) [23]. According to the Monro-Kellie theory, headaches may also be related to painful venous and arterial cerebral vasodilation, which compensates for decreased CSF volume [3, 21]. In the inner ear, hypotension of the perilymph also causes an imbalance between endolymph and perilymph itself, thereby leading to hearing impairment, tinnitus and/or vertigo [16, 24].

In most cases, dural puncture closes spontaneously without consequence. PDPH is reported to resolve in more than 50% of patients within 4 days, and in more than 70% within a week. However, headache may persist in a limited number of patients [3, 20]. PDPH can limit patients’ activity, increase the duration of hospital stay and use of care [25] and is particularly disabling during the postpartum period, when the mother has to take care of her child. It is therefore important to treat this syndrome. Epidural blood patch is currently the gold standard and most effective treatment for PDPH. However, the timing for this treatment remains uncertain. Some studies have suggested a greater risk of failure when administering an early blood patch, although no causal link could be established [26–28]. The diminished effectiveness in this case, could be explained by the severity of the CSF leak [28]. Conversely, administering a delayed blood patch could increase bed rest duration. A delay of 24 to 36 h between the onset of headaches and the completion of a blood patch seems reasonable. However, delaying the blood patch by more than 48 h is not recommended [29].

Historically, the reported success rate after the first blood patch is approximately 80% [28, 30]. Recently, however, efficacy has been found to be lower (32% at most) [27]. Moreover, it is an invasive procedure that may result in rare but serious complications such as spinal subdural hematoma [31] or permanent paraparesis and cauda equina syndrome [32]. It may also trigger anxiety and discomfort in patients and can sometimes be painful (back pain, transient bradycardia).

Alternative treatments have recently been studied; they aim to be less invasive or to reduce headache intensity whilst waiting for the blood patch or the disappearance of PDPH. Several lines of evidence have suggested the effectiveness of adrenocorticotropic hormone (ACTH) in the management of PDPH [33–41]. Several mechanisms have been proposed to explain the effects of ACTH or its analogues on headaches. First, it has been shown by several teams that fragments of ACTH interact with opioid receptors *in vitro* and have morphine-like effects *in vivo* [42–44]. Second, ACTH may increase brain β-endorphins which can change the perception of pain [35–37]. Third, ACTH stimulates the adrenal cortex, which releases different hormones such as glucocorticoids, androgens and mineralocorticoids. Glucocorticoids have an anti-inflammatory effect that may explain in part the analgesia observed after ACTH injection [38]. Fourth, mineralocorticoids are responsible for fluid retention that may lead to meningeal oedema and overlapping edges of the dural puncture [36].

The trial reported by Hakim found that administration of tetracosactide after accidental dural puncture in 90 parturients was associated with a significant reduction in the incidence of PDPH and requirement for epidural blood patch [39]. The efficacy of tetracosactide for the treatment of PDPH in 32 patients has been reported to be 56% (95% CI [33; 79%]) [40], and to be similar to that of epidural blood patch in a study that included 28 patients [41], although 4 of the 15 in the tetracosactide group also received a blood patch. However, a randomized trial that included 18 patients did not find any efficacy of tetracosactide in the treatment of PDPH, nor in the reduction of blood patch use [45]. This study was conducted among a small group of patients and did not report any data on the interval between PDPH onset and blood patch. Taken together, efficacy evidence of ACTH analogues on PDPH remains limited, with contradictory results obtained in small groups of patients. Therefore, drawing conclusions and establishing a protocol for the management of PDPH are difficult.

In the proposed study, we have designed a therapeutic trial to evaluate the efficacy of tetracosactide in the treatment of PDPH in 88 postpartum patients.

## Methods/Design

### Aim of the study

The aim of this study is to assess the efficacy and safety of tetracosactide (Synacthen®) in the treatment of post-dural puncture syndrome for patients who received neuraxial anaesthesia during childbirth.

Our working hypothesis is that a single intravenous infusion of tetracosactide may avoid the need for the epidural blood patch. To challenge this hypothesis two groups will be compared: the study group receiving an infusion of 1 mg of tetracosactide and the control group receiving a placebo (0.9% saline). Both groups receive standard analgesic treatment and epidural blood patch if needed (after a minimum of 24 h following the injection of the experimental treatment).

### Trial design

The study is a randomised, double-blind, placebo-controlled, parallel-arm, double-centre trial. It is conducted in two different sites, and both sites are part of the same French university hospital. We followed the SPIRIT checklist (Additional file 1). Patient enrolment started in October 2016 and was expected to be completed within 2 years after the start of the study. However, as explained in section 1.3.11. this did not happen.

Once participants are enrolled in the study with informed consent, they are randomised into the two study arms. The study group receives 1 mg of tetracosactide intravenously whereas the control group receives 0.9% saline. Outcomes will be measured between 2 and 6 h, on day 1, day 2, day 3, and between days 13 and 17 post-randomisation. Adverse events are collected throughout the study.

### Primary endpoint

The primary endpoint of the study is the rate of epidural blood patch within a 15-day follow-up period, that is, the number of patients who receive a blood patch within the 15-day period divided by the total number of participants included.

### Secondary endpoints

**Fig. 1:**
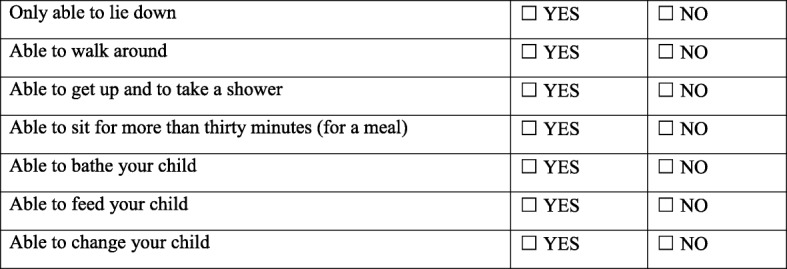
Questionnaire for qualitative assessment of activity limitation: a simple questionnaire corresponding to the needs of a postpartum patient

The secondary endpoints are as follows:

- duration of headache, that is, the mean number of days with headache within the 15-day follow-up period
- intensity of headache (11-point numerical rating scale, NRS) [46], that is, the mean NRS at day 1, 2, 3, and 15
- the need for analgesic treatment (descriptive analysis of type, time interval after study treatment initiation, and duration) within the 15-day follow-up period
- appearance of associated features (neck stiffness, tinnitus, hypoacusia, photophobia, diplopia, dizziness, or nausea), that is, a descriptive analysis of these features within the 15-day follow-up period
- activity limitation (self-assessed, both qualitatively as described in Fig. 1, and quantitatively on a scale from 0 to 100%), with a mean score give on days 1, 2, 3 and 15
- length of hospital stay, that is the mean number of days spent in hospital assessed from inclusion to day 15
- the number of epidural blood patches performed per patient, that is, the mean number within the 15-day follow-up period
- tolerance, assessed by the collection of all adverse events (type, frequency, and severity)

### Population

#### Number of patients needed

The sample size calculation is based on a previous study that investigated tetracosactide in PDPH [39] and, retrospectively, on observational data obtained from our hospital database. The primary endpoint is the rate of blood patch in the two groups. We defined the efficacy of tetracosactide as a decrease in blood patch use by 30% in the tetracosactide group, a clinically relevant difference reducing the need for invasive treatment and replacing it with an easily administered treatment that has a known safety profile.

In our centre, 90% of patients with PDPH after epidural analgesia receive a blood patch. The study reported by Hakim [39] found a greater than 50% decrease in PDPH incidence and blood patch use after prophylactic treatment with tetracosactide, in a context of epidural analgesia. We therefore need to include 44 subjects in each group, given a power of 0.80 and type I error of 0.025 (α = 2.5% Fisher’s exact test, power of 80%, p1 = 0.9; p2 = 0.6), to reduce the blood patch requirement by 30%. Thus a total of 88 patients need to be randomised.

#### Eligibility

Patients eligible for enrolment in this clinical trial are those suffering from PDPH due to epidural analgesia, combined spinal-epidural analgesia, or spinal anaesthesia for childbirth.

The headache characteristics should be as follows:

- within 5 days after delivery
- relieved in the supine position and/or worse while sitting or standing
- intense (NRS > 3/10)
- with or without: neck stiffness, tinnitus, hypoacusia, photophobia, visual impairment, nausea or vomiting
- after having eliminated differential diagnosis for pre-eclampsia or eclampsia, cerebral venous thrombosis, migraine.

Patients must be at least 18 years old and benefit from healthcare insurance. They also have to provide a written informed consent.

#### Exclusion criteria

Exclusion criteria are as follows:

- diplopia (which requires an epidural blood patch without delay)
- contraindication to ACTH: uncontrolled arterial hypertension, uncontrolled diabetes mellitus, acute psychosis, infectious diseases
- contraindication to tetracosactide:
  - ○ currently receiving a drug associated with an increased risk of Torsades de Pointes (astemizole, bepridil, IV erythromycin, halofantrine, pentamidine, sparfloxacine, sultopride, terfenadine or vincamine)
  - ○ patient received a live vaccine in the month prior to inclusion
- previous history of hypersensitivity to tetracosactide
- patient who has received tetracosactide since childbirth
- contraindication to epidural blood patch: HIV, HVC, leukocytosis, fever
- pre-eclampsia or eclampsia during the current pregnancy (may be confounding for the aetiology of headache)
- patient who has already received a prophylactic blood patch (at the time of accidental dural puncture diagnosis)
- Under 18 years old or adult under guardianship
- Mental disorder which does not allow informed consent
- Patients who are enrolled in another clinical trial

#### Consent

When a diagnosis of PDPH is made by the anaesthesiologist, screening of patients for inclusion and exclusion criteria is performed. The investigator then presents the study to the patients. Written information is provided to the patients. Patients are given some time to think before committing to the study. Final inclusion occurs shortly after they have read the information letter, raised questions they may have and signed the written informed consent.

### Randomisation, blinding, and data management

The anaesthesiologist checks the patient’s inclusion criteria and the absence of exclusion criteria. After signing of the informed consent, patients are randomised online using the ClinSight-Online software program (Ennov society, Paris, France). The randomisation list was compiled by a statistician, entered into the software and communicated to the pharmacist to prepare the numbered treatment kits. The randomization is performed by block stratification according to site. Patients are allocated to a treatment number (by the ClinSight software). The study is performed double-blinded; both patients and the medical team are blinded to treatment allocation. An anaesthetic nurse prepares the treatment according to the assigned number to which the rest of the healthcare team is blinded. Patient data are collected on electronic case report forms (eCRFs). The sponsor will independently monitor both locations of this multisite trial every ten patients. This data monitoring is performed by a clinical research associate (CRA). His/her role is to check the consent form, the quality of data collection (missing data, data entry errors …), the availability of treatment at each site, the pharmacy process, and the declaration of any serious adverse event. He/she is employed by the sponsor but is independent of the investigators and is not involved in carrying out the study or interpretation of the results. Anonymized data are only accessible to the investigators, the sponsor, and other authorized persons (statistician, data manager …). They will be stored confidentially for 15 years by the sponsor. They will not be used for further studies without further patient agreement. At the end of the study, we plan to communicate trial results to participants, healthcare professionals and the public via a peer-reviewed publication. Authors will be considered in the case of a substantial contribution to the conception or design of the study or the acquisition, analysis or interpretation of data. They will need to have drafted the work or revised it critically. They will all approve the final version to be published. We do not expect the participation of a professional writer. Data not disclosed in the publication will not be accessible.

### Intervention protocols

During the post-partum period, patients are usually followed by midwives. In case of headache, whether an accidental dural puncture is diagnosed or not, the anaesthesiologist is informed by the midwives. Therefore, any symptomatic patient is examined by an anaesthesiologist. The study investigators are the anaesthesiologists working in the maternity department of the two centres.

Patients are enrolled when diagnosed with PDPH based on the anaesthesiologist’s clinical evaluation. They are randomised to receive intravenous tetracosactide or placebo. In the study group, patients receive a single intravenous injection of 1 mg tetracosactide (Synacthen®, Sigma-Tau laboratory, Roma, Italy). Four vials containing 0.25 mg tetracosactide (in 1 ml of solvent) are reconstituted in 100 ml of normal saline (0.9% NaCl). This solution is infused intravenously over 20 min. In the control group, patients receive an equal volume of normal saline: 104 ml over 20 min. The whole intervention is administered over a 20-min course and therefore is not modifiable once begun except for the occurrence of a side effect. A side effect is defined herein as any new abnormality observed upon clinical examination, occurring during the injection. In this case, the treatment is immediately stopped but monitoring and data collection continues.

Standard analgesic medication, as concomitant treatments, is also initiated in both groups from the beginning of headache, as follows:

- for mild headache (NRS < 3/10): 1 g paracetamol and 400 mg ibuprofen orally every 6 h
- for moderate headache (NRS = 3): nefopam 20 mg and/or a combination of paracetamol 300 mg and opium 10 mg is given in addition every 4 h
- for severe headache (NRS ≥ 4): morphine 10 mg orally every 4 h can be added as required

These treatments are adjusted daily.

**Fig. 2:**
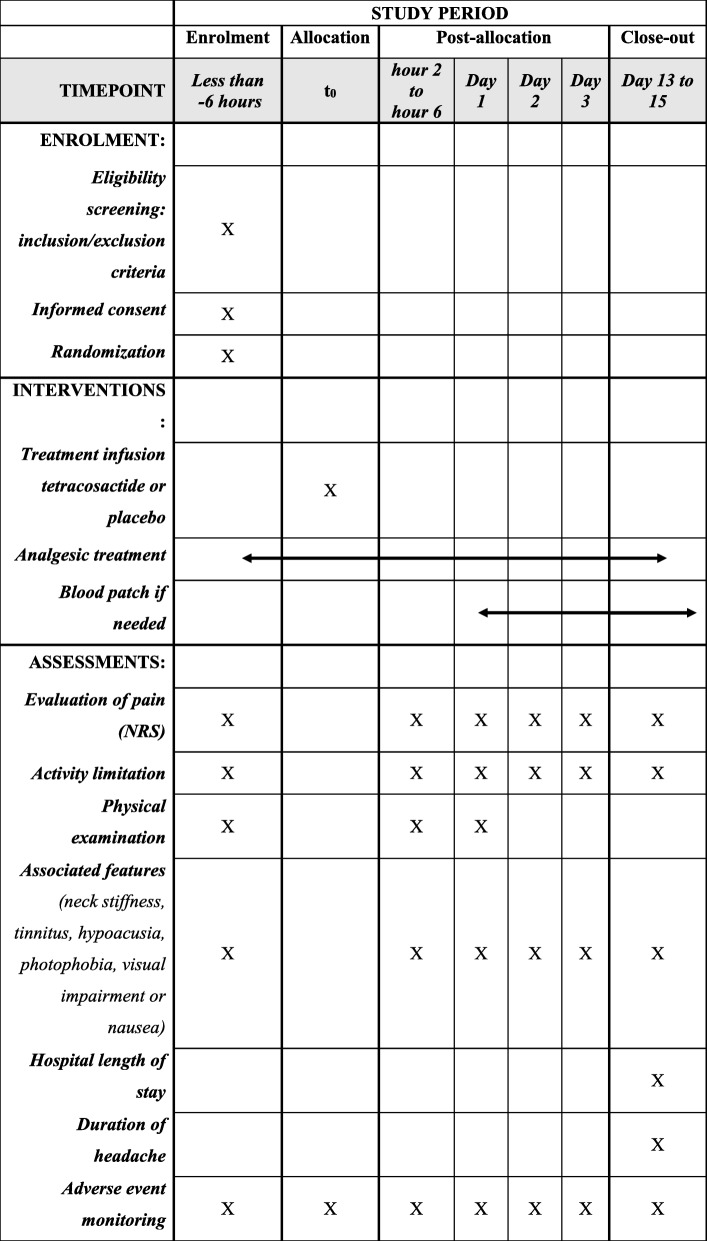
Schedule of enrolment, intervention and assessment

The primary and secondary endpoint, including tolerance criteria, are evaluated blinded during infusion of the study treatment, 2–6 h later, 1 day, 2 days, 3 days and between 13 and 17 days post-treatment by the investigator. The latter is conducted by phone call in order to increase the complete follow-up. The total duration of the study is therefore 13 to 17 days. Any alternative analgesic intervention required after receiving the study treatment is reported at each time point as indicated above. The study timeline is summarised in Fig. 2.

Epidural blood patch is performed for patients who reported a persistent moderate to severe headache a minimum of 24 h after administration of the study treatment and 36 h after the dural puncture, based on the clinical appreciation of the anaesthesiologist. Epidural blood patch can be repeated twice if required (i.e., if the headache does not improve or if it increases again). The anaesthesiologist performs the epidural blood patch. Autologous blood is injected into the epidural space using an 18-gauge Tuohy needle. The injection is stopped when the patient feels pressure in the lower back or pain.

### Statistical analysis

Unblinding occurs at the end of the study and once the database has been frozen. Analyses will be carried out on an intention-to-treat (ITT) basis. If data are missing from visits, we will carry forward the last value for which the pain (and other symptoms) has not improved. This is the most pessimistic scenario for this condition, which usually improves spontaneously. Blood patch can only be performed at the hospital, and it is therefore very unlikely that there will be missing data regarding the primary endpoint. If a patient does not receive full treatment, she will be included in the group to which she was randomised for further ITT analysis. Statistical analyses will be performed using IBM SPSS statistics for windows (IBM corp., Armonk, NY, USA) and R (R Foundation for Statistical Computing, Vienna, Austria). All data will be checked for normal distribution using the Kolmogorov test. For normally distributed data, variables will be presented as mean ± standard deviation (SD). Non-normally distributed data will be presented as the median and interquartile range [IQR]. Categorical variables will be presented as number and percentage of the total. To compare the two groups (tetracosactide and placebo), we will use Fisher’s exact test for qualitative data and the non-parametric Mann-Whitney tests for quantitative variables. A comparison of the two groups at randomisation will identify potential bias due to unequal allocation. A multivariate logistic regression analysis will be performed to study factors independently associated with the response to tetracosactide and to control potential confounding factors.

#### Intermediate analysis

The hypothesis of a 30% difference between the two groups is conservative compared to the 50% reduction reported by Hakim [39]; a difference of only 30% between the two groups should already be clinically relevant. An interim analysis is therefore planned after the enrolment of 44 patients. The overall alpha risk will be adjusted according to the Bonferroni method and will be 2.5% (5% / 2). A *p* value < 0.025 will therefore be considered statistically significant. If this initial analysis is statistically significant (*p* < 0.025), the independent monitoring committee may decide to stop the study without waiting for the planned recruitment. Otherwise, the study will be continued until the inclusion of 88 patients.

### Ethical approval

This trial is conducted in accordance with the protocol and in compliance with the moral, ethical and scientific principles governing clinical research as set out in the Declaration of Helsinki (1989) and Good Clinical Practice (GCP). It is also conducted in accordance with French legislation (Public Health Code; Act No. 2004–806 of 9 August 2004). It is registered in the Clinical Trial Protocol Registration and Results System: NCT02813655 (registration date: 24 June 2016). An ethics committee (*Comité de Protection des Personnes sud Est V*, CPP) has approved the study for both participating sites on 6 April 2016. The national drugs authority (*Agence Nationale de Sécurité du Médicament et des Produits de Santé*, ANSM) authorised this trial (authorisation date: 18 April 2016).

### Adverse events management

During the study period, adverse events will be reported and recorded in the participants’ CRF. Adverse events include any new abnormality observed upon clinical examination, occurring during the study treatment injection or the follow-up period, or any allergic reaction to the treatment. The severity of adverse events will be graded as mild, moderate, severe, life threatening, or death, and the potential relationship between the adverse event and the study treatment will be assessed by clinical judgment. If an adverse event requiring hospitalisation extension or causing a medically critical situation occurs, the event will be recorded as a severe adverse event. All severe and life threatening adverse events or death will be reported to the investigator and to the sponsor within 24 h after the information has been collected. The sponsor will report all such events to legal authorities (ANSM and CPP). The investigator must follow patients until the adverse event has been resolved. All adverse events collected will be described in the trial publication. A severe adverse event is the only reason for unblinding participants and providers before the end of the study. It will be performed by the poison control centre (Anti-poison Centre) which is independent of the study and is the only party with access to the randomisation list.

### Protocol amendment

Because patient enrolment/inclusion was more difficult than expected, the protocol was amended (January 2017) to extend inclusion from PDPH due to a dural puncture carried out by large Tuohy needle to all postpartum PDPH, i.e. PDPH related to a large dural puncture and PDPH related to small needle (25-27G) for spinal anaesthesia. For this reason, a second amendment (November 2018) extended the inclusion period until October 10, 2021.

## Discussion

We propose herein a protocol to investigate the use of tetracosactide, an ACTH analogue, to treat PDPH. Blood-patch is currently the gold standard for PDPH treatment, and tetracosactide could be interesting as it would reduce the need for the invasive procedure and replace it with an easily administered treatment that has a known safety profile. Given previous results obtained in small groups of patients, evidence for the efficacy of ACTH analogues for PDPH treatment remains limited.

Initially, the inclusion was limited to PDPH due to a dural puncture carried out by a large Tuohy needle. Because patient enrolment/inclusion was more difficult than expected, the protocol was amended to extend inclusion to all postpartum PDPH. This widening of the inclusion criteria raises the issue of population heterogeneity, which we hope to limit by the randomisation process.

Since the management strategy of PDPH (using blood-patch and non-invasive treatments) is not clearly defined, the rate of blood-patch varies. Rucklidge, for example, described that 50 blood-patches were received by 144 patients treated for PDPH after epidural anaesthesia over 20 years in the hospital in which he practiced [47]; the blood-patch rate was therefore 35%. In the study reported by Aya et al. all patients (100%) treated for PDPH after epidural obstetric anaesthesia in their unit received a blood patch [48]. Therefore, a potential limitation of the study described herein is that the sample size could be insufficient if the rate of blood patch in the control group is lower than 90%.

## Conclusion

We expect a decrease in the use of epidural blood patch in those receiving tetracosactide, thus indicating a decrease in PDPH symptoms for these patients. This will define the therapeutic success of tetracosactide and the possibility to use this treatment as a non-invasive alternative to blood patch for PDPH treatment.

## Trial status

The fourth protocol version dated 28 September 28 is in place. The study is ongoing; the first patient was included in October 2016, and in May 2019, 25 patients had been enrolled. Initially we expected to finalise the study in October 2018, but we had to extend the inclusion phase until 2021 to reach a total of 88 inclusions. Due to the ongoing nature of the study and to avoid influencing its conduct, the final results are not presented herein. An intermediate analysis will be performed after 44 inclusions, and if a significant difference is observed between the two groups concerning the primary outcome, the study will be discontinued.

## Supplementary information

**Additional file 1.** SPIRIT 2013 Checklist: Recommended items to address in a clinical trial protocol and related documents*.

## Data Availability

Data sharing is not applicable to this article as no datasets have been generated or analysed yet during the current study.

## Abbreviations

ACTH: Adrenocorticotropic hormone
ANSM: *Agence Nationale de Sécurité du Médicament et des Produits de Santé*
CPP: *Comité de Protection des Personnes*
CRA: Clinical research associate
CSF: Cerebrospinal fluid
eCRFs: Electronic case report forms
GCP: Good clinical practice
HCV: Hepatitis c virus
HIV: Human immunodeficiency virus
IQR: Interquartile range
ITT: Intention-to-treat
NRS: Numerical rating scale
PDPH: Post-dural puncture headache
SD: Standard deviation
